# Basics in hip chondrolabral lesions and state of the art

**DOI:** 10.1051/sicotj/2017040

**Published:** 2017-12-22

**Authors:** Mohamed Abd El-Radi, Oliver R. Marin-Peña, Hatem Galal Said, Marc Tey-Pons

**Affiliations:** 1 Orthopedic Surgery and Traumatology, University Hospital Assuit Assiut Egypt; 2 Orthopedic Surgery and Traumatology, University Hospital Infanta Leonor Madrid Spain; 3 Orthopedic Surgery and Traumatology, University Hospital del Mar Barcelona Spain

**Keywords:** Hip joint, Cartilage, Chondrolabral junction, Arthroscopy, Treatment options

## Abstract

Chondrolabral complex is a weak point along an histological transition zone. Most cartilage and labral lesions in the femoroacetabular impingement syndrome are located in this area. Different classifications are used to evaluate the severity and predict the prognosis of chondrolabral complex injuries. Acetabular Labrum Articular Disruption (ALAD) and Multicenter Arthroscopy of the Hip Outcomes Research Network (MAHORN) classifications are commonly used with a prognosis and treatment implication. Treatment of chondrolabral lesions detected on magnetic resonance imaging (MRI), should only be considered when clinical symptoms are presented. A wide range of treatment options include debridement with or without microfracture, repair or regenerate therapies. The future of hip joint preservation should be directed towards to the development of the treatment of chondrolabral injuries.

## Anatomy

The hip joint is a deeply seated weight-bearing articulation and has an important concern with anatomical stresses in comparison with the shoulder joint. It contains cartilage of two forms: hyaline cartilage, from the union of osteochondral complexes developed in gestational life, and labral fibrocartilage [1–3]. The hip joint also contains the ligamentum teres, which has an early developmental role in formation of the articulation as well as a structural role [4–6]. A fibrocartilagenous tissue, named the labrum, is situated all around the bony acetabular rim and it adds to the stability and congruity of the joint. It has the remarkable role of providing a tight biological seal that protects the articular cartilage [3, 7, 8].

**Figure 1.**
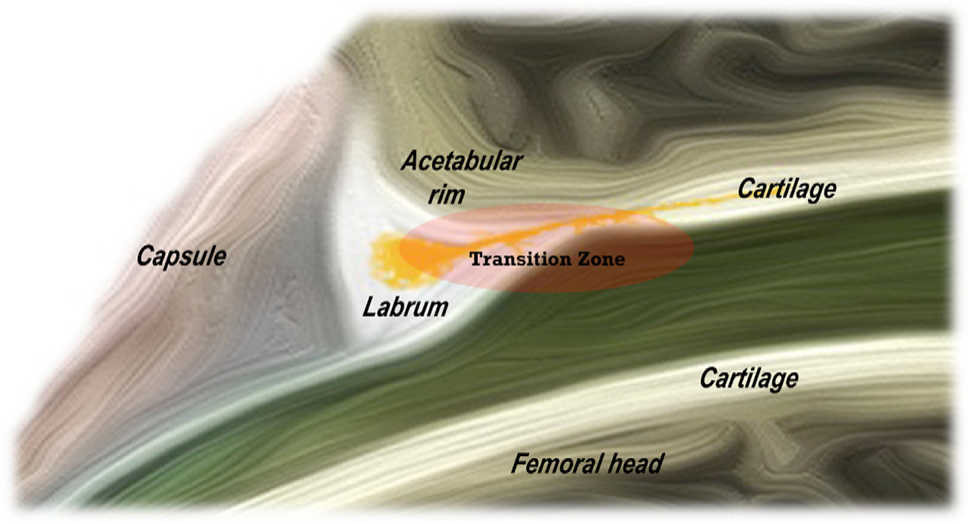
Diagram showing the transition zone of chondrolabral complex.

The chondrolabral complex structure has an intrinsic weak point or a transition zone between the acetabular labrum and the acetabular articular cartilage, especially in the anterior segment (Figure 1) [9].

## Pathophysiology

**Figure 2.**
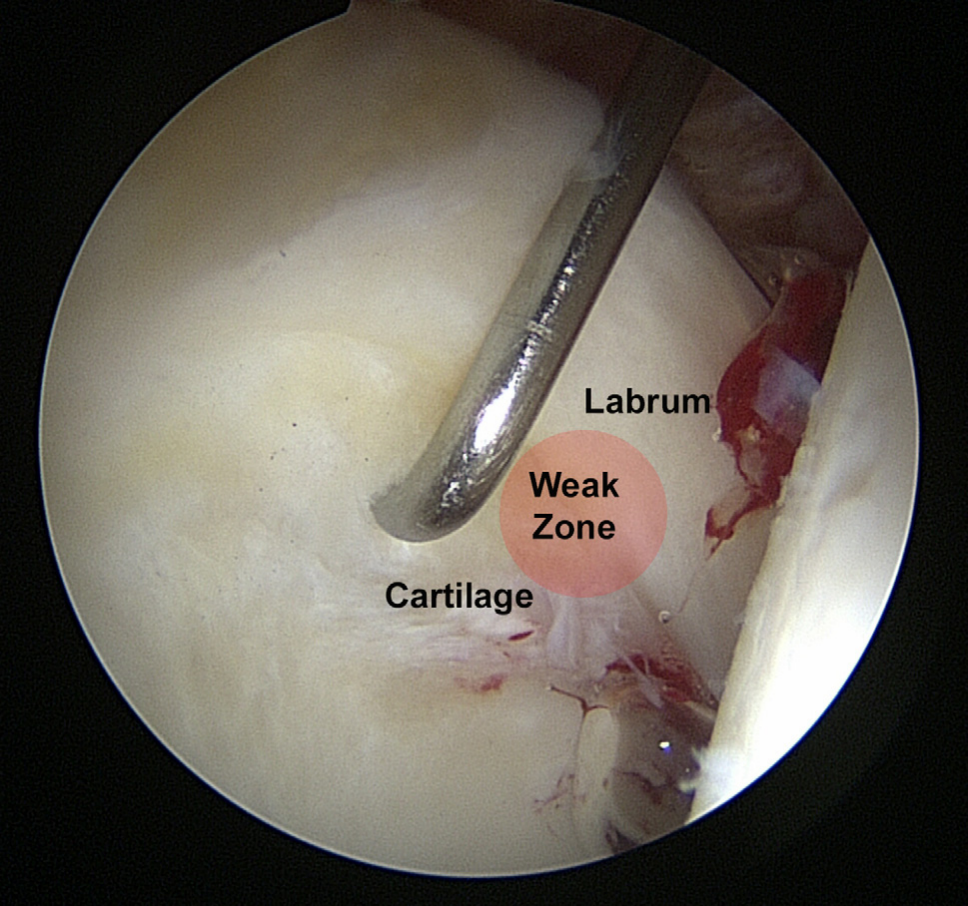
Damage of the chondrolabral transition (“weak zone”) in a cam FAI.

In normal hips, the acetabular labrum fuses with the articular cartilage of the acetabular side through the transition zone smoothly without any defects [10]. One reason for chondrolabral junction injuries affecting adults is the impingement syndrome. Femoroacetabular impingement (FAI) has two types, which are cam type and pincer type. The cam type impingement is encountered in the pathomechanical analysis of injury to the chondrolabral complex and is produced by a primary osseous variant of the head-neck junction at the femoral head epiphyseal line, but it can be secondary to several known causes, such as Perthes disease, epiphyseal slipping of the femoral head, or following fractures of the femoral neck; it might be considered as an idiopathic entity [11–13]. In the cam type impingement, the articular cartilage of the acetabulum is affected anterosuperiorly. The labrum is attached firmly to the underlying bone, but the articular cartilage is peeled from the labrum at this weak point of transition [14] (Figure 2). The main problem in the cam FAI type is the absence of a waist area at the femoral head-neck junction. In flexion phases, this part is compressed against the acetabulum anterosuperiorly and this generates shear force at that transition point and in subchondral bone. This causes the labrum to be stretched and pushed laterally, and the cartilage is pushed centrally [15]. In hips with the pincer type, the cartilage is usually affected circumferentially and that causes damage to the acetabular cartilage at its anterosuperior junctional margin. In that situation, the generated force causes the labrum to be compressed between the femoral head at one side and underlying bone at the other side which limits the predicted injury at the rim zone of the acetabular cartilage [14].

## Classifications

Different classification systems assess the severity and predict the prognosis of chondrolabral complex injuries.

Beck’s Classification [16]:

- Malacia: roughening of surface, fibrillation.
- Debonding: loss of fixation to the subchondral bone, carpet phenomenon.
- Cleavage: loss of fixation to the subchondral bone, frayed edges, cartilage thinning, flap.
- Defect: complete thickness defect.

**Figure 3.**
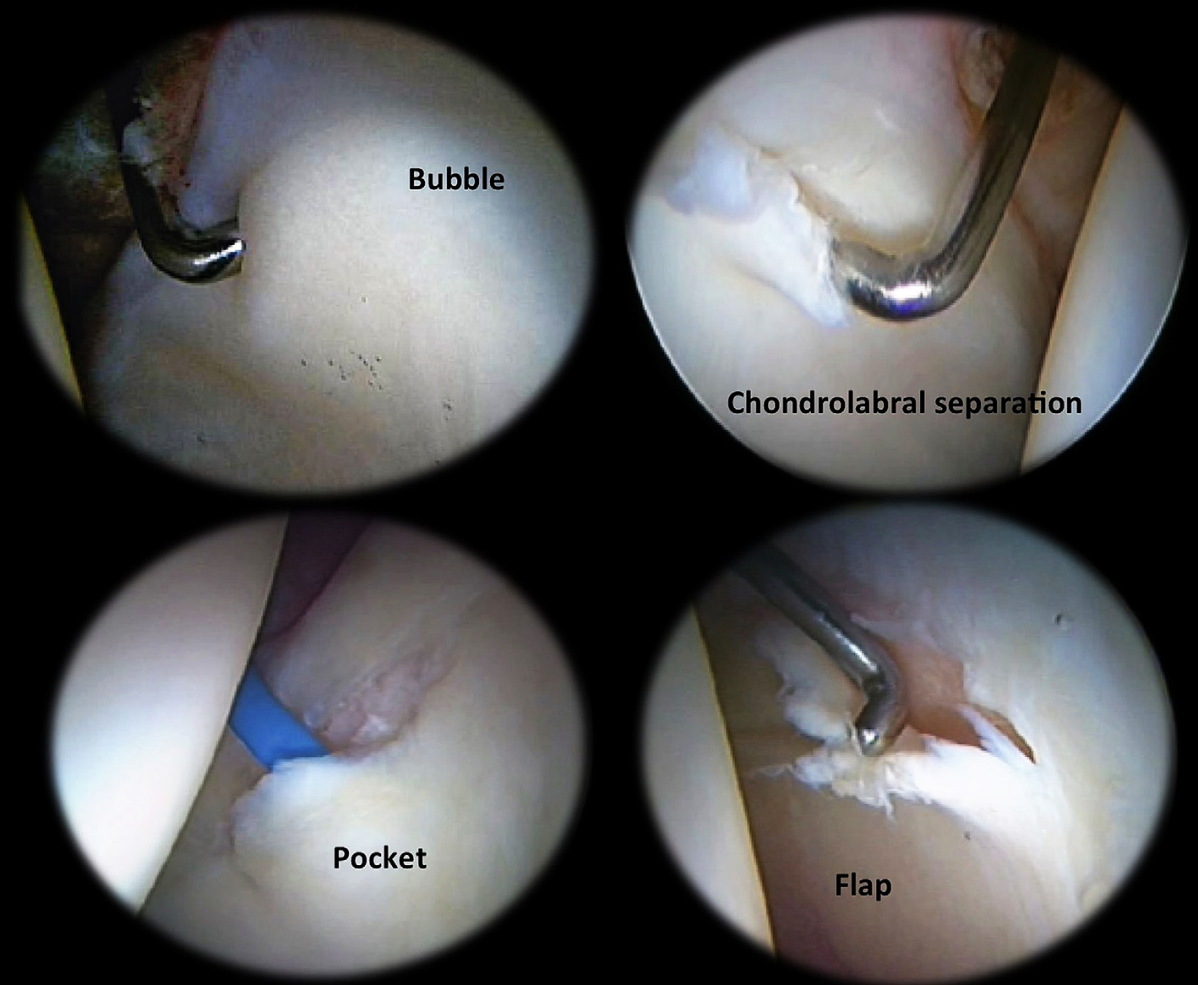
MAHORN classification. Bubble (upper left), separation (upper right), pocket (bottom left), flap (bottom right).

MAHORN Classification [17] (Figure 3):

- Bubble: this is a palpable bulkiness to the articular cartilage at the periphery. This lesion probably represents delamination of the articular cartilage and is sometimes referred to as “wave sign”.
- Chondrolabral separation: shearing forces cause chondrolabral separation at its junctional zone.
- Pocket: when the delamination of a bubble connects to a chondrolabral separation tear, a pocket is formed.
- Flap: once the pocket becomes unstable. It is the commonest finding in the arthroscopy of cam-type femoroacetabular impingement.
- Defect: the final step is loss of the flap by disintegration or detachment.

Acetabular Labrum Articular Disruption (ALAD) Classification [18]:

- ALAD 1: softening of the adjacent cartilage.
- ALAD 2: early peel of the cartilage (carpet delamination).
- ALAD 3: large flap of the cartilage.
- ALAD 4: loss of cartilage.

**Figure 4.**
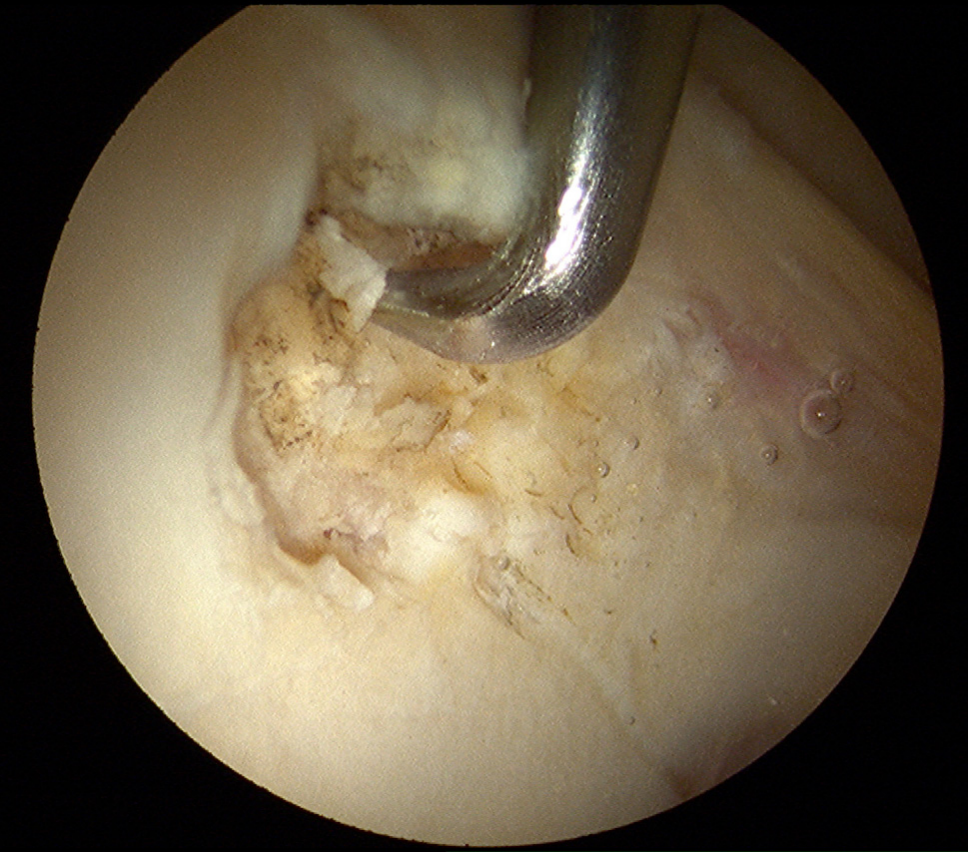
Cartilage lesion ALAD 4 treated with debridement and stabilization of the borders together with microfracturing.

If we compare ALAD and MAHORN classifications, lesion type ALAD 2 could be similar to a “bubble” in the MAHORN classification (Figure 3). Otherwise, ALAD 3 cartilage injury correlates with flap lesion in MAHORN (Figure 4).

## Treatment options

**Figure 5.**
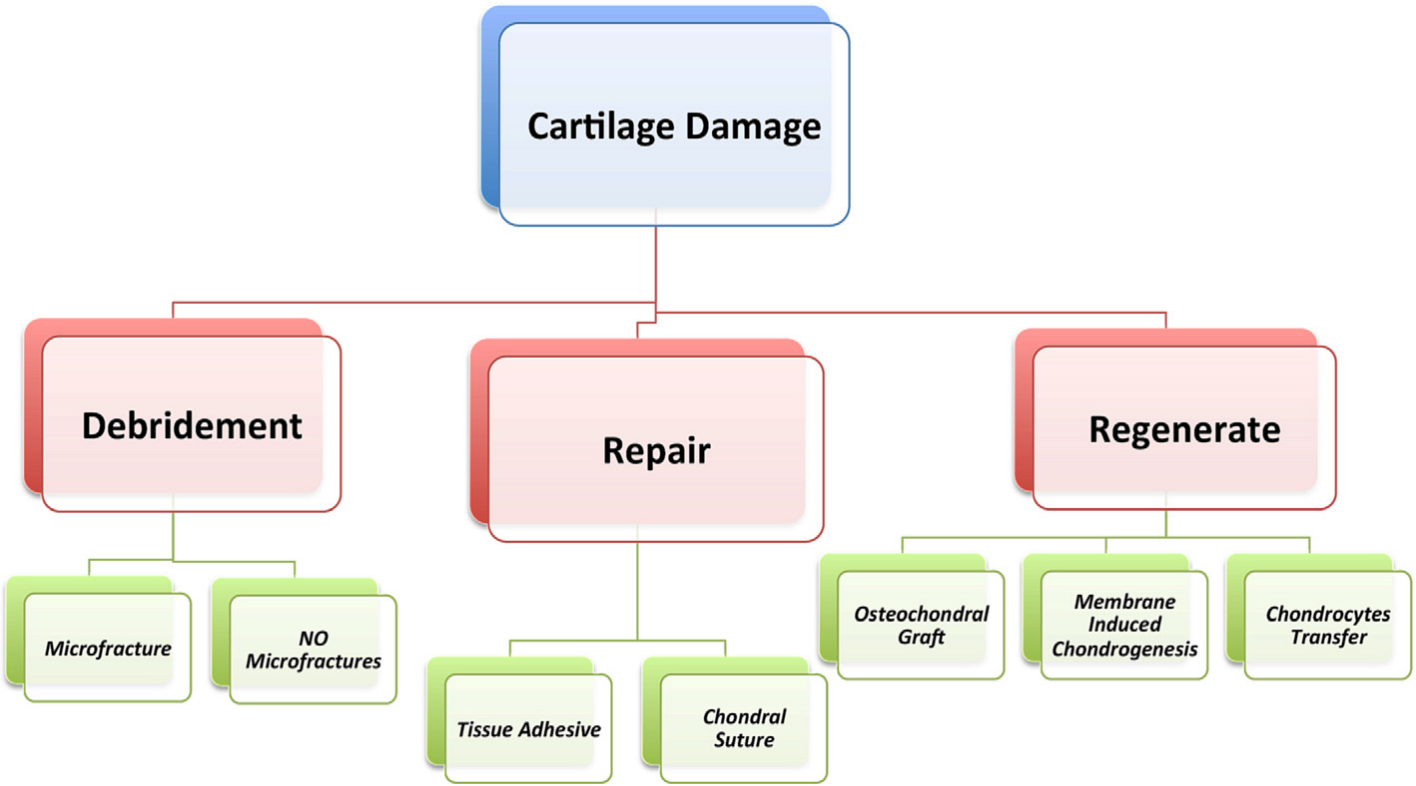
Chondrolabral lesions management.

Treatment of such injuries detected on MRI, should only be considered when clinical symptoms are present. Clinical symptoms and signs of chondrolabral lesions are variable. Femoroacetabular lesions usually present with ill-defined groin pain on the affected side [19, 20]. This pain is related directly to mechanical situations especially with flexion and internal rotation [14]. Correct imaging techniques ensure the exact extent of the chondrolabral lesion is seen. Treatment of chondrolabral injuries is dependent on the time of patient presentation. The gold standard treatment of an arthritic hip is total joint replacement [21–23]. Recently, many articular cartilage strategies have been tried to restore focal and diffuse damage in the active patient (Figure 5). The use of microfracture techniques is described to restore focal articular cartilage defects through stimulation of inner pelvic wall stem cells [23–26]. The replacement cartilage in these defects is found to have some characteristics of the hyaline cartilage [25]. Philippon et al. [27] evaluated, through a second look hip arthroscopy, the filling percentage of chondral defects following the microfracture procedure at the acetabular side in the nine patients. They reported the percentage of filling as high as 95–100% in injuries at a mean of 20-month follow-up [27]. Domb et al. [28] examined the clinical improvement in a cohort study looking at the patient reported outcome at two-year follow-up. They demonstrated excellent results after the microfracture technique. McDonald et al. [24] and Fontana et al. [29] reported that athletes competing at high level, returned to their previous level of activity after they were treated by the microfracture technique. Cartilage regeneration techniques were studied by Fontana et al. [29]. They documented the comparison between autologous chondrocyte transplantation and simple debridement in an equal number of patients with chondral defect of grade three or four of Outerbridge classification at the acetabular articular cartilage. They demonstrated an improved clinical outcome in chondrocyte transplantation patients at 74 months postoperatively in comparison to the other group.

## Conclusions

Chondrolabral injuries continue to be a challenge for hip arthroscopists. Nevertheless, many new treatment alternatives have been developing in the last few years and clinical results of these techniques will be published in the following decade. The future of hip joint preservation should be improved by the further development of the treatment of chondrolabral injuries.

## Conflict of interest

The authors declare that they have no conflict of interest in relation with this paper.
